# Xeroderma Pigmentosum: Gene Variants and Splice Variants

**DOI:** 10.3390/genes12081173

**Published:** 2021-07-29

**Authors:** Marie Christine Martens, Steffen Emmert, Lars Boeckmann

**Affiliations:** Clinic and Policlinic for Dermatology and Venerology, University Medical Center Rostock, 18057 Rostock, Germany; christine.martens2@med.uni-rostock.de (M.C.M.); steffen.emmert@med.uni-rostock.de (S.E.)

**Keywords:** Xeroderma pigmentosum, nucleotide excision repair, gene variants, alternative splicing, precision medicine

## Abstract

The nucleotide excision repair (NER) is essential for the repair of ultraviolet (UV)-induced DNA damage, such as cyclobutane pyrimidine dimers (CPDs) and 6,4-pyrimidine-pyrimidone dimers (6,4-PPs). Alterations in genes of the NER can lead to DNA damage repair disorders such as Xeroderma pigmentosum (XP). XP is a rare autosomal recessive genetic disorder associated with UV-sensitivity and early onset of skin cancer. Recently, extensive research has been conducted on the functional relevance of splice variants and their relation to cancer. Here, we focus on the functional relevance of alternative splice variants of XP genes.

## 1. Introduction

Ultraviolet (UV) radiation can cause direct and indirect DNA damage and has long been recognized as the primary cause of photocarcinogenesis. Because UV-B radiation (280–315 nm) can be directly absorbed by DNA, it has a high mutagenic potential. UV-B radiation leads to the formation of cyclobutane pyrimidine dimers (CPDs) and 6,4-pyrimidine-pyrimidone dimers (6,4-PPs) within the DNA, so-called bulky lesions that distort the DNA backbone [1]. The nucleotide excision repair (NER) is essential for the repair of ultraviolet (UV)-associated DNA lesions. It can recognize DNA damage in the entire genome (global genome = GG, GG-NER) and during transcription (transcription coupled = TC, TC-NER). While the initial recognition process is different between GG-NER and TC-NER, the subsequent steps of the NER process are the same: unwinding of the DNA by helicases, dual incision of the damaged DNA strand by endonucleases to remove the lesion and DNA synthesis with ligation to fill the gap [2].

More than 40 genes are involved in the NER. Several clinical entities result from NER repair defects, such as Xeroderma pigmentosum (XP; MIM 278700, 610651, 278720, 278730, 278740, 278760, 278780), Cockayne syndrome (CS; MIM 216400, 133540), trichothiodystrophy (TTD; MIM 601675, 616390, 616395) and cerebro-oculo-facio-skeletal-syndrome (COFS; MIM 214150, 610756, 616570, 610758). Interestingly, different mutations in the multifunctional genes of the NER—namely XPB, XPD, XPF and XPG—can lead to different clinical entities or overlap syndromes [3,4]. In this review, we provide an overview of XP as a clinical entity and recent research about spontaneously occurring alternative splice variants of XP genes and their significance for DNA repair and cancer risk.

## 2. Xeroderma Pigmentosum

Xeroderma pigmentosum (XP) is a rare autosomal recessive genetic disorder with a worldwide prevalence of 1:1,000,000. The underlying genetic defects can be assigned to seven complementation groups—XP-A to XP-G—and one variant form (XPV). While the variant form affects the translesional DNA polymerase η, the affected genes of the seven complementation groups are all involved in the nucleotide excision repair (NER) [5].

**Figure 1:**
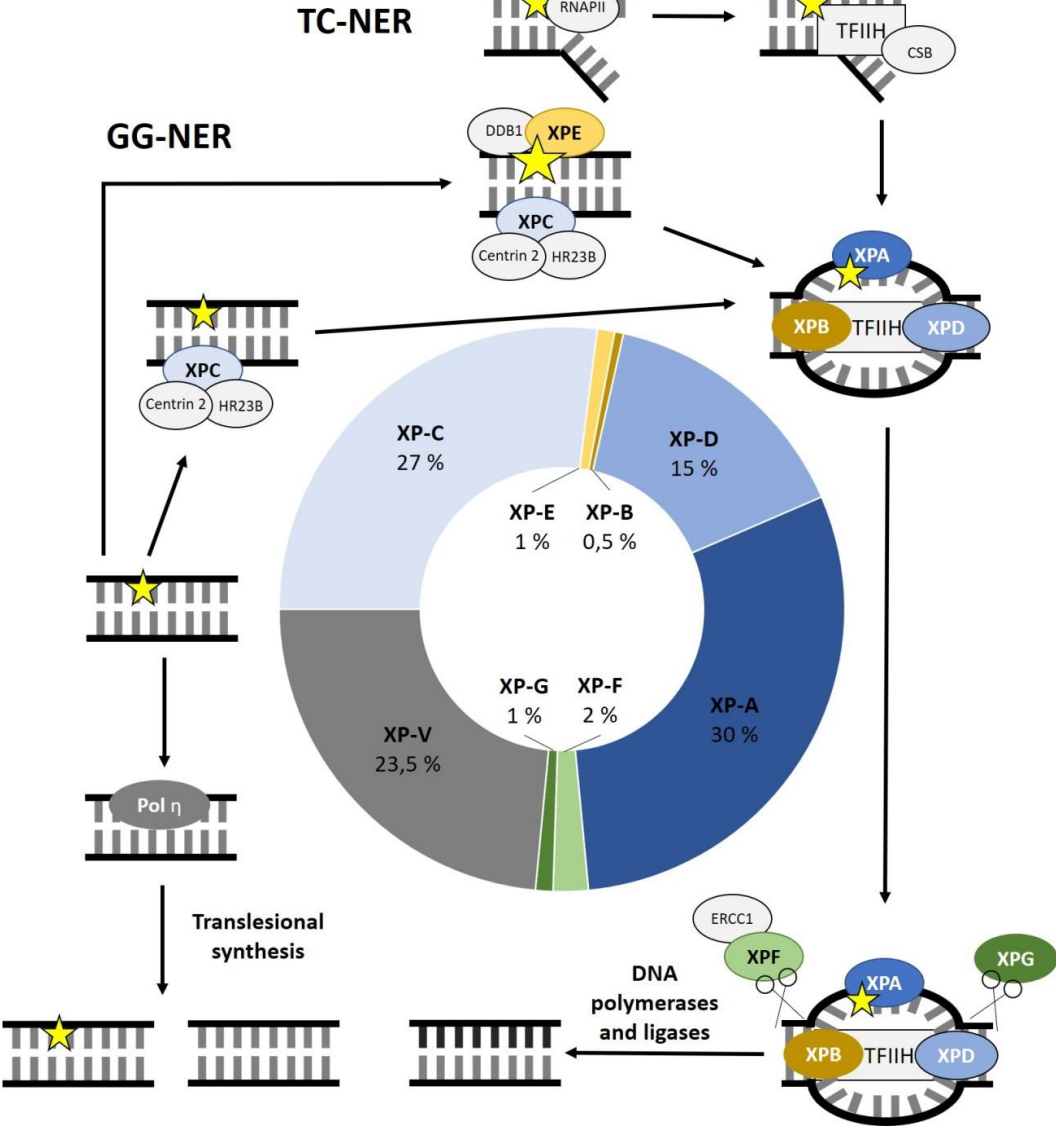
Overview of the nucleotide excision repair (NER) process and frequencies of genes affected in Xeroderma pigmentosum (XP). Global genome (GG)-NER relies on the XPC complex sensing distortions within the DNA due to DNA damage. HR23B and centrin 1 are supposed to increase the activity of XPC and the stability of the XPC complex respectively. To better identify minor distortions in the DNA, the UV-DDB complex, consisting of DDB1 and XPE, induces a more recognizable kink in the DNA. Transcription-coupled (TC)-NER is initiated when the RNA polymerase II (RNAPII) is stalled due to DNA damage. This leads to the recruitment of CSA, CSB and UVSSA, which in turn recruit the transcription factor II H (TFIIH) complex. The DNA helicases XPB and XPD are two of ten protein subunits of the TFIIH complex. They open the double-stranded DNA around the lesion. Afterwards, the endonucleases XPF-ERCC1 and XPG excise the damaged, single-stranded DNA, leaving a gap that is soon filled by DNA polymerases and DNA ligases. XPA has been reported to be important for incision coordination as well as identification of single-stranded DNA damage. Patients with XP variant (XPV) have a mutation in the translesional DNA polymerase η. The diagram in the middle shows the frequency of the affected complementation groups in XP.

Out of the seven complementation groups, some genes are more frequently affected than others. XP-A is the most commonly affected complementation group, representing 30% of all XP patients, followed by XP-C with 27%. XP-D accounts for 15%. Defects in XP-F, XP-E and XP-G are less common with 2%, 1% and 1%, respectively. With 0.5% of XP cases, XP-B is the least frequently affected complementation group. Defects in XPV make up 23.5% of all cases (Figure 1) [6]. Certain geographic regions show a higher prevalence of XP cases. This can be attributed to isolation, cultural influences, or less mobility. For example, the prevalence of XP is 1 to 22,000 in Japan. A founder mutation in *XPA* can be found in almost 1% of the Japanese population. Other founder mutations were reported in *XPC* in the Northern African population and in *XPD* in Iraqi families of Jewish decent [7].

### 2.1. Xeroderma Pigmentosum and the Nucleotide Excision Repair

During GG-NER, the DNA lesion is recognized by the XPC complex, which senses distortions within the DNA. It consists of the following three subunits: XPC, HR23B and centrin 2. XPC preferentially binds to the undamaged stretch of complementary DNA opposite the lesion. HR23B is said to increase the activity of XPC in NER, while centrin 2 stabilizes the complex. If there is only a modest distortion within the DNA due to the lesion, the UV-DDB complex, consisting of DDB1 and XPE (=DDB2), can help to identify damaged DNA. It induces a more recognizable kink in the DNA so that the XPC complex can identify the lesion more easily. XPC is a substrate for the transcription factor II H (TFIIH) complex [2,8].

For TC-NER, the DNA lesion is recognized by the RNA polymerase II (RNAPII) during transcription. The stalled RNAPII initiates TC-NER, leading to the recruitment of multiple proteins such as CSA (=ERCC8), CSB (=ERCC6) and UVSSA. In turn, these proteins recruit the TFIIH complex [2,9].

In both pathways, DNA damage recognition leads to the recruitment of the TFIIH complex. The TFIIH core complex consists of seven protein subunits including the two helicase subunits XPB (=ERCC3) and XPD (=ERCC2). These helicases open the DNA double strand around the lesion having opposite polarities. The single-stranded, damaged DNA segment is then excised by the endonucleases XPF (=ERCC4)-ERCC1 and XPG (=ERCC5), acting 5′ and 3′ of the lesion, respectively. Among other functions, XPA can identify DNA damage on the single-stranded DNA (ssRNA) and is important for the incision coordination. After the excision, the 22–30 nucleotide long gap is filled by DNA polymerases (DNA Polymerase δ, DNA Polymerase ε or DNA Polymerase κ) and DNA ligases (DNA ligase 1 or XRCC-DNA ligase 3). Figure 1 shows the main steps of the NER outlining the function of XP-related proteins and the frequencies of genes affected in XP (Figure 1) [2,10,11].

### 2.2. Xeroderma Pigmentosum: Clinical Manifestation

Only about 60% of XP patients suffer from increased sun sensitivity. Nevertheless, all XP patients show early hyperpigmentation of sun exposed skin and early onset of premature skin ageing. Some XP patients may also suffer from poikiloderma at birth, but not hyperpigmentation. Due to the defective NER, UV-induced DNA damage accumulates, leading to skin cancer at an early age [7]. The average age of XP patients developing skin cancer is 8 years old compared to 60 years old in the general population [12]. Neurological symptoms occur in approximately 25% of all XP patients, ranging from missing tendon reflexes to speech disturbances, ataxia, peripheral neuropathy, cognitive decline and loss of the ability to walk or talk. Neurological degeneration is primarily associated with patients with mutations in *XPA* and *XPD* but has been reported for patients with mutations in *XPB*, *XPG* and *XPF,* as well. Patients with neurological symptoms have a lower life expectancy than XP patients without neurological symptoms [13,14]. A rare and severe form of XP with neurological symptoms is the De Sanctis–Cacchione syndrome (MIM 278800). In addition to severe XP with neurological symptoms, these patients exhibit a short stature and hypogonadism [15]. Overlapping syndromes with other NER disorders such as CS, TTD and COFS can also extend the symptomatic spectrum of XP with symptoms of the overlapping NER disorder [3]. Interestingly, the severity of clinical symptoms of XP patients varies among complementation groups. It also seems to depend on the location of the genetic alteration within the gene of the complementation group. To understand the link of these genetic variations and the associated clinical phenotypes, worldwide cooperation to study genotype–phenotype correlations has been proposed due to the rarity of XP cases [16,17].

### 2.3. Xeroderma Pigmentosum: Diagnostics

Generally, XP is a clinical diagnosis. The patient should be assessed interdisciplinarily by dermatologists, ophthalmologists, ENT, neurologists, radiologists and human geneticists [5]. In addition to the clinical diagnosis, functional tests, gene and protein expression analysis as well as sequence analysis are available to identify the affected gene. For example, the host cell reactivation assay (HCR) provides a functional assessment of the DNA repair capability of the patient cells. For this assay, a previously UV-irradiated plasmid containing a luciferase gene is transfected into the patient cells. Due to the reduced NER, luciferase expression in patient cells is reduced compared to wild-type (WT) cells. To determine the complementation group, expression plasmids of XP genes are co-transfected, leading to the repair of UV-induced DNA damage and therefore higher luciferase expression [18]. While these complementation assays are the reason why we have so-called complementation groups, HCR assays have been used for functional testing of patient cells to determine their DNA repair capability. Sequencing of the genes involved, especially next generation sequencing (NGS), has been shown to be a fast and effective diagnostic tool [19].

### 2.4. Xeroderma Pigmentosum: Therapy and Prevention

XP cannot be cured, only treated. To prevent or delay symptoms, an early diagnosis is essential, so that the patients can take protective measures such as systematic sun protection (e.g., sunscreen, long-sleeved clothing, broad-brimmed hats, sunglasses, facial protection, window foils, UV meters) and quarterly dermatologic skin cancer screenings. Skin cancers should be treated according to standard therapy guidelines, with surgery being the treatment of choice for invasive skin cancer. Due to the number of surgeries required, excisions on the face should be as small as possible. Other treatment options include the use of imiquimod 5% cream for basal cell carcinomas, the SMO (smoothened) inhibitor vismodegib, the PD-1 (programmed cell death-1) inhibitor pembrolizumab and systemic retinoids [7].

## 3. Splice Variants of Xeroderma Pigmentosum Genes

Splicing and alternative splicing occurs in the cells of every human being [20]. Splicing defines the process of removing the non-coding introns from pre-mRNA and joining the coding exons together. Alternative splicing describes alternative ways for processing of the pre-mRNA. After transcription, the newly formed pre-mRNA, containing both introns and exons, is processed into the final mRNA, which is then translated into a protein. During alternative splicing, exons can be skipped, alternative splice sites can be selected, introns can be retained in the final mRNA and exons can be mutually exclusive. Additionally, transcription can be modified, so that multiple pre-mRNAs can be produced from one gene. Transcript variants and splice variants can lead to either alternative proteins or non-coding transcripts. Alternative proteins deriving from one gene are called isoforms [21,22]. Protein isoforms can have identical or completely opposing functions [23]. With regard to spontaneous splice variants of XP genes, we focus on isoforms with residual DNA repair capability and the expression of the truncated protein. Our hypothesis is that a high expression of such splice variants enhances cellular DNA repair capability and therefore postpone cancer development. On the contrary, a high expression of splice variants with only residual repair capability could negatively affect the WT repair capability and overall reduce the cellular repair capacity, leading to accelerated tumorigenesis. Thirdly, they could not have any function at all due to, e.g., loss of a nuclear localization signal (NLS) despite measurable truncated protein expression in the cell.

A disturbed splicing regulation can be found in cancer, leading to a more dominant expression of alternative splice variants [24]. For example, a spontaneous splice variant of the androgen receptor (AR)—AR splice variant 7 (AR-V7)—has been associated with castration-resistant prostate carcinoma. Lacking the ligand binding domain, it retains its ability to bind to DNA and activate transcription [25,26]. Another example is an alternative splice variant of CD44, a cell surface receptor involved in cell survival and proliferation. The variant CD44v6 has been shown to be overexpressed in a variety of cancers. In a mouse model, CD44v6-directed Chimeric Antigen Receptor modified (CAR) T cell therapy showed promising results in the treatment of solid tumors [27,28,29,30].

Additionally, mutation of splice sites can create pathogenic splice variants [18]. A resistance mechanism to RAF inhibitors has been discovered in melanoma involving an isoform of BRAF (V600E). This isoform lacks the exons that code for the RAS-binding domain and dimerizes in a RAS-independent manner, enabling downstream signaling [31]. To deal with this resistance mechanism, dimer inhibition is under investigation. Ponatinib, a kinase inhibitor, has been shown to be a promising candidate for the development of new treatments of BRAF-dependent tumors [32]. Interestingly, considering XP an alternative splice variant of an altered allele has been shown to be beneficial in a patient with a mutation in *XPG,* leading to a less severe form of XP/CS [33].

Recently, our group has been focusing on spontaneous splice variants of XP genes and their impact on DNA repair. For all XP genes, putative spontaneous splice variants are listed in databases such as Ensembl (https://www.ensembl.org/index.html, accessed on 7 June 2021). These putative splice variants can be verified, and further splice variants can be identified by sequencing using reverse transcriptase polymerase chain reaction to produce cDNA. These splice variants can then be cloned into expression vectors. For functional assessment, immortalized cells are needed that are deficient of the respective XP gene.

Several genome editing methods are available to produce such a cell line. While zinc finger nucleases (ZFN), transcription activator-like effector nucleases (TALEN) and meganucleases can be used to create knockout cell lines, they are inefficient, expensive and time-consuming methods [34]. The innovative clustered regularly interspaced short palindromic repeats (CRISPR)/CRISPR-associated (Cas) nuclease 9 (CRISPR/Cas9) system is a valuable tool for genome editing. As opposed to the aforementioned methods, it uses an RNA sequence to lead the endonuclease to the targeted DNA sequence. While the CRISPR RNA (crRNA) identifies the target, a trans-activating crRNA (tracrRNA) is needed for endonuclease activity. To simplify the method and to make it more efficient, a single guide RNA (sgRNA) was created as a combination of the crRNA and tracrRNA. The endonuclease Cas9 induces a double-stranded break (DSB) in the DNA. This DSB can be repaired by either homology-directed repair (HDR) or non-homologous end joining (NHEJ). While HDR repairs the DSB using a repair template, NHEJ is error-prone. It regulates the DSB, but often produces insertion/deletion (indel) mutations, resulting in frameshifts and premature stop codons [35,36]. Previously, we were able to show that lentiviral transduction was more efficient than transient transfection to generate *SNAP29* knockout human fibroblast cell lines. Although lentiviral transduction efficiency was higher, genome integration and therefore cumulating off-target effects and insertional mutation need to be considered [37].

We also created CRISPR/Cas9-mediated *XPF* knockout cells. Using transient transfection, we studied the function of spontaneous splice variants of *XPF* in those cells. XPF consists of 916 amino acids (aa). There are three main functional domains: the SF2 helicase-like domain, a nuclease domain and a helix–hairpin–helix motif. Two putative NLS have been detected within the SF2 helicase-like domain. Three isoforms of *XPF* were detected and analyzed. All *XPF* splice variants were able to enter the nucleus. While the isoform XPF-201 lacks only the first 12 aa, XPF-003 is severely truncated, containing only the first 372 aa. The isoform XPF-202 lacks both the N-terminal 12 aa and is C-terminally truncated like XPF-003. The two severely truncated isoforms lack the nuclease domain and the helix–hairpin–helix motif. Isoforms XPF-201 and XPF-003 exhibited residual repair capabilities. Due to XPF-202 not displaying any significant repair capabilities, the first 12 amino acids seem to be essential for protein function. Interestingly, when overexpressed, all XPF splice variants as well as the full-length protein reduced NER capabilities [38,39].

To analyze the function of *XPG* alternative splice variants, primary fibroblasts from an XP-G patient (XP20BE) were used. XPG consists of 1186 aa. The catalytic center of the protein is formed by two domains, separated by a spacer domain. Three putative NLS have been identified: one at the end of the first part of the nuclease domain and the other two close to the C-terminus. For *XPG,* seven isoforms have been identified. All *XPG* splice variants were able to enter the nucleus. Five isoforms are N-terminally truncated: XPG IsoII (142 aa), XPG IsoIII (232 aa), XPG IsoIV (302 aa), XPG IsoV (663 aa) and XPG IsoVI (763 aa). None of these include the second domain of the catalytic center. The isoform XPG-201 lacks the first 168 aa and thus the first domain of the catalytic center. This is a deletion of exon 1–4. The isoform XPG-202 is a conjoined product containing the immunoglobulin-like variant motif (484 aa) and lacking exon 1 of *XPG*, including the first domain of the catalytic center. This leads to a protein of 1676 aa. Residual NER capabilities were reported for the isoforms XPG IsoV and XPG IsoVI. Additionally, overexpression of XPG-201 and XPG IsoVI inhibited NER capabilities. Interestingly, alternative splice variant expression levels of XPG isoforms varied between different tissues and between individuals [39,40].

Three isoforms have been reported for *XPC* that lead to exon skipping of either exon 4, 7 or 12. A single nucleotide polymorphism (SNP) associated with exon 12 skipping has been shown to produce an isoform with reduced DNA repair function. Additionally, a dominant negative effect for this isoform on NER has been shown in WT cells [41]. A variety of SNP in XP genes have also been reported to be associated with cancer risk, depending on ethnicity and cancer type. These include but are not limited to the following examples: An A/G polymorphism in *XPA* (rs1800975) was shown to have an impact on overall cancer and skin cancer susceptibility in the Caucasian population. An *XPC* PAT+/− polymorphism was suggested to influence the susceptibility to prostate cancer and may increase melanoma and head and neck squamous cell carcinoma risk. Carriers of the rs13181 polymorphism in *XPD* have been shown to have a higher lung cancer, esophageal cancer, acute myeloid leukemia and glioma risk, but not ovarian cancer risk. It has also been shown to be likely associated with a higher risk of colorectal cancer in the Indian population and a higher risk of digestive tract cancers in Asian populations. The polymorphism rs873601 in *XPG* has been associated with overall cancer risk [42,43,44,45,46,47,48,49,50,51,52].

Since abnormal function of DNA repair pathways is associated with the initiation and progression of cancer [53], further research should be conducted on the influence of alternative splice variants of XP genes on cancer risk. If a correlation between certain splice variants and cancer risk could be verified, these findings could be used to establish a marker panel to determine the individual cancer risk. It could also include gene variants such as the aforementioned SNP. Such a marker panel could be useful to identify high-risk patients who need more frequent preventative check-ups.

Further studies are needed to evaluate the capacity of splice variants of XP genes as cancer risk markers.

## 4. Conclusions

Due to its impaired DNA damage repair of UV-induced lesions, XP can be used as a basis to further study the molecular mechanism of NER and photocarcinogenesis. Previous studies showed CRISPR/Cas9-mediated knockout cells of XP genes as a viable tool to study the functions of XP genes. Additionally, the functional relevance of spontaneous alternative splice variants of XP genes can be evaluated.

Identification and characterization of alternative splice variants of XP-related genes may help to identify patients with an increased risk for cancer development and thus provide novel biomarkers for better precision medicine. This warrants more research and clinical correlation studies.

## Data Availability

The data presented in this review are available in the references following each paragraph.
